# Parameter-Dependency of Low-Intensity Vibration for Wound Healing in Diabetic Mice

**DOI:** 10.3389/fbioe.2021.654920

**Published:** 2021-03-09

**Authors:** Rita E. Roberts, Onur Bilgen, Rhonda D. Kineman, Timothy J. Koh

**Affiliations:** ^1^Department of Kinesiology and Nutrition, University of Illinois at Chicago, Chicago, IL, United States; ^2^Center for Tissue Repair and Regeneration, University of Illinois at Chicago, Chicago, IL, United States; ^3^Jesse Brown VA Medical Center, Chicago, IL, United States; ^4^Department of Mechanical and Aerospace Engineering, Rutgers University, Piscataway, NJ, United States; ^5^Department of Medicine, Section of Endocrinology, Diabetes and Metabolism, University of Illinois at Chicago, Chicago, IL, United States

**Keywords:** low-intensity vibration, wound healing, angiogenesis, growth factors, diabetes

## Abstract

Chronic wounds in diabetic patients represent an escalating health problem, leading to significant morbidity and mortality. Our group previously reported that whole body low-intensity vibration (LIV) can improve angiogenesis and wound healing in diabetic mice. The purpose of the current study was to determine whether effects of LIV on wound healing are frequency and/or amplitude dependent. Wound healing was assessed in diabetic (db/db) mice exposed to one of four LIV protocols with different combinations of two acceleration magnitudes (0.3 and 0.6 g) and two frequencies (45 and 90 Hz) or in non-vibration sham controls. The low acceleration, low frequency protocol (0.3 g and 45 Hz) was the only one that improved wound healing, increasing angiogenesis and granulation tissue formation, leading to accelerated re-epithelialization and wound closure. Other protocols had little to no impact on healing with some evidence that 0.6 g accelerations negatively affected wound closure. The 0.3 g, 45 Hz protocol also increased levels of insulin-like growth factor-1 and tended to increase levels of vascular endothelial growth factor in wounds, but had no effect on levels of basic fibroblast growth factor or platelet derived growth factor-bb, indicating that this LIV protocol induces specific growth factors during wound healing. Our findings demonstrate parameter-dependent effects of LIV for improving wound healing that can be exploited for future mechanistic and therapeutic studies.

## Introduction

Chronic wounds represent an escalating health problem around the world, especially in diabetic patients. Over 415 million people (8.3% of the world’s adult population) are afflicted with diabetes and associated complications, including chronic wounds (Chatterjee et al., 2017). People with diabetes incur a 25% lifetime risk of developing chronic wounds, which often lead to amputation, resulting in decreased quality of life, high morbidity and mortality (Ramsey et al., 1999; Jeffcoate and Harding, 2003; Hoffstad et al., 2015). Wound healing typically occurs through overlapping phases of inflammation, proliferation and remodeling (Koh and DiPietro, 2011; Eming et al., 2014). Although chronic wounds are known to exhibit defects in each phase of healing, including dysregulated inflammation, impaired perfusion and neovascularization, and poor tissue maturation (Blakytny and Jude, 2006), few therapies are available to improve healing of diabetic wounds.

Energy-based modalities are often used in conjunction with standard treatments for hard to heal chronic wounds. These treatments use laser, electrical, or mechanical stimulation, in an attempt to modify the cellular and biochemical environment to improve angiogenesis and healing (Ennis et al., 2016; Game et al., 2016; Sousa and Batista Kde, 2016). Recently, our group demonstrated that whole body low-intensity vibration (LIV) can improve angiogenesis and wound healing in diabetic mice, potentially by increasing growth factors such as insulin-like growth factor (IGF)-1 and vascular endothelial growth factor (VEGF) in the wound (Weinheimer-Haus et al., 2014). In addition, we and others have demonstrated that LIV can rapidly increase systemic and regional (i.e., skin) blood flow (Nakagami et al., 2007; Maloney-Hinds et al., 2008; Tzen et al., 2018; Zhu et al., 2020) and can inhibit progression of pressure ulcers (Arashi et al., 2010; Sari et al., 2015). However, much remains to be learned about how LIV signals influence different aspects of wound healing.

The purpose of the current study was to identify LIV amplitudes and frequencies that promote healing in diabetic mice. The hypothesis of this study was that effects of LIV on wound healing are frequency and amplitude dependent.

## Materials and Methods

### Animals

Diabetic db/db mice (BKS.Cg-Dock7m+/+Leprdb/J) were obtained from the Jackson Laboratory. Experiments were performed on 12–16 weeks-old male mice. Only mice with fasting blood glucose >250 mg/dl were included in the study. Mice were housed in environmentally controlled conditions with a 12-h light/dark cycle. Water and food were available *ad libitum*. Two wounds from each of four mice were analyzed for each assay (*N* = 8 total for each assay). To minimize bias, mice were randomly assigned to experimental groups and resulting samples were coded and analyzed in a blinded fashion. All procedures involving animals were approved by the Animal Care Committee at the Jesse Brown Veterans’ Affairs Medical Center [OLAW Assurance number D16-00722 (A4456-01)].

### Excisional Wounding

Mice were subjected to excisional wounding as described previously (Weinheimer-Haus et al., 2014). Briefly, mice were anesthetized with isoflurane and their dorsum was shaved and cleaned with alcohol. Four 8 mm wounds were made on the back of each mouse with a dermal biopsy punch and covered with Tegaderm (3M, Minneapolis, MN, United States) to keep the wounds moist and maintain consistency with treatment of human wounds.

### Low-Intensity Vibration

Following wounding, mice were randomly assigned to one of four whole-body LIV treatment groups or to a non-vibration sham (control) group. LIV treatment groups utilized different combinations of low (45 Hz) and high (90 Hz) frequencies and low (0.3 g) and high (0.6 g) peak accelerations. Harmonic LIV signals were calibrated using an accelerometer attached directly to top surface of the vibrating plate (Weinheimer-Haus et al., 2014). For LIV treatment, mice were placed in an empty cage directly on a vibrating plate, and LIV was applied for 30 min per day for 7 days/week starting on the day of wounding [cf (Weinheimer-Haus et al., 2014) for image of set-up]. Non-vibrated sham controls were similarly placed in a separate empty cage but were not subjected to LIV.

### Wound Closure

Wound closure was assessed in digital images of the external wound surface taken immediately after injury and on days 3, 6, and 10 post-injury. Wound area was measured using Fiji Image J and expressed as a percentage of the area immediately after injury.

### Wound Histology

Skin wounds were collected from the pelt of each animal on day 10 post-injury, followed by embedding in tissue freezing medium and freezing in isopentane cooled with dry ice. Each wound was cryosectioned from one edge to well past the center and 10-μm sections were selected from the center of the wound for staining and analysis of re-epithelialization, granulation tissue formation, angiogenesis and collagen deposition (Weinheimer-Haus et al., 2014). For all wound healing analyses, digital images were obtained using a Keyence BZ-X710 microscope with 2×/0.10 or 20×/0.75 Nikon objectives and BZ-X Analyzer software.

#### Re-Epithelialization and Granulation Tissue Area

Wound re-epithelialization and granulation tissue area were assessed by morphometric analysis of hematoxylin and eosin stained cryosections from the wound center (Weinheimer-Haus et al., 2014). The distance between the wound edges, defined by the distance between the first hair follicle encountered at each end of the wound, and the distance that the epithelium had traversed into the wound, were measured using image analysis software. Re-epithelialization was then calculated as: [(distance traversed by epithelium)/(distance between wound edges) × 100]. Granulation tissue area was measured as the area of new tissue formation between wound edges. Re-epithelialization and granulation tissue area were measured in three sections per wound and was averaged over sections to provide a representative value for each wound.

#### Angiogenesis and Collagen Deposition

Dermal healing was assessed using immunohistochemical staining for platelet-derived endothelial cell adhesion molecule-1 (also called CD31) for angiogenesis and Masson’s trichrome stain for collagen deposition (Weinheimer-Haus et al., 2014). For angiogenesis assessment, sections were first air-dried, fixed in cold acetone, washed with PBS, quenched with 0.3% hydrogen peroxide, then washed again with PBS. Sections were blocked with buffer containing 3% bovine serum albumin and then incubated overnight with CD31 antibody (1:100, Biolegend, San Diego, CA, United States). Sections were then washed with PBS and incubated with biotinylated anti-rat secondary antibody (1:200, Vector Laboratories, Burlingame, CA, United States). After a wash with PBS, sections were incubated with avidin D-horseradish peroxidase (1:1000) and developed with a 3-amino-9-ethylcarbazole kit (Vector Laboratories). Image J was used to quantify the percentage of CD31-stained area relative to the total area of the wound bed. For each assay, digital images covering the majority of the wound bed (usually three images at ×20 magnification) were first obtained. The percent area stained in each image was then quantified by counting the number of pixels staining above a threshold intensity and normalizing to the total number of pixels. Threshold intensity was set such that only clearly stained pixels were counted. The software allowed the observer to exclude staining identified as artifact, large vessels, and areas deemed to be outside the wound bed. For trichrome analysis, staining was performed according to the manufacturer’s directions (IMEB, San Marcos, CA, United States), and Image J was used to quantify the percentage of blue collagen-stained area relative to the total area of the wound bed. For both trichrome and CD31 staining, three sections per wound were analyzed, and data were averaged over sections to provide a representative value for each wound.

### ELISA

Wounds were snap frozen and stored in LN2 and then homogenized in cold PBS (10 μl of PBS per mg wound tissue) supplemented with protease inhibitor cocktail (Sigma Aldrich, St. Louis, MO, United States) using a dounce homogenizer and then centrifuged. Supernatants were used for enzyme-linked immunoassay of IGF-1, VEGF, basic fibroblast growth factor (bFGF) and platelet derived growth factor (PDGF)-bb (R&D Systems, Minneapolis, MN, United States).

### Statistics

Values are reported as means ± standard deviation. Measurements of wound healing or wound growth factors were compared between treatment groups using one-way ANOVA. Dunnett’s multiple comparisons test was used when ANOVAs demonstrated significance. Differences between groups were considered significant if *P* ≤ 0.05.

## Results

### LIV Promotes Wound Closure in a Parameter Dependent Fashion

**FIGURE 1:**
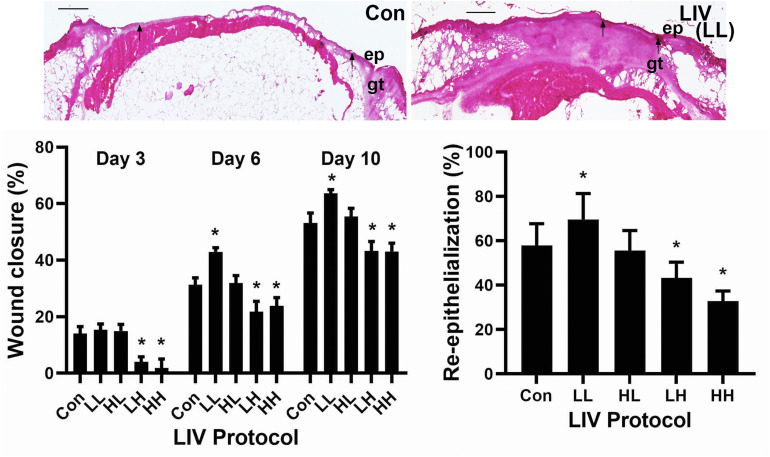
Parameter dependency of low-intensity vibration (LIV) for improving wound closure. Mice either received one of four protocols of whole-body LIV: 0.3 g at 45 Hz (LL), 0.3 g at 90 Hz (LH), 1.0 g at 45 Hz (HL), 1.0 g at 90 Hz (HH) or sham control treatment (Con) for 30 min per day, starting the day of wounding for 5 days per week. Left: Wound closure was measured in digital images of wound surface on days 3, 6, and 10 post-injury. Right: Re-epithelialization was measured in hematoxylin and eosin stained sections from center of day 10 wounds. Top: Representative images of hematoxylin and eosin stained sections; arrows mark ends of epithelial tongues growing into wound, ep = epithelium, gt = granulation tissue, scale bar = 500 μm. Two wounds from each of four mice were analyzed for each assay (*N* = 8 total for each assay). *Mean value significantly different from that of Con for same time point, *P* ≤ 0.05.

Mice were treated with one of four LIV protocols: low (45 Hz) frequency, low (0.3 g) acceleration (LL), high (90 Hz) frequency, high (0.6 g) acceleration (HH), low frequency, high acceleration (LH) or high frequency, low acceleration (HL) (Table 1). Consistent with our previous study (Weinheimer-Haus et al., 2014), none of the protocols in the current study altered blood glucose levels of the diabetic db/db mice (data not shown). The LL protocol was the only one to increase external measurements of wound closure on days 6 and 10 post-injury compared to sham control, whereas the LH and HH protocols decreased wound closure at all time points (Figure 1). Similarly, the LL protocol was the only one to increase histological measurements of re-epithelialization on day 10 post-injury compared to sham control, whereas the LH and HH protocols decreased re-epithelialization (Figure 1). In short, the LL protocol improved wound closure in diabetic mice, whereas the LH and HH protocols impaired wound closure.

**TABLE 1:** LIV protocols.

	LL	LH	HL	HH
Acceleration (g)	0.3	0.6	0.3	0.6
Frequency (Hz)	45	45	90	90

*LL, low acceleration, low frequency; LH, low acceleration, high frequency; HL, high acceleration, low frequency; HH, high acceleration, high frequency.*

### LIV Promotes Wound Angiogenesis and Granulation Tissue Formation in a Parameter Dependent Fashion

**FIGURE 2:**
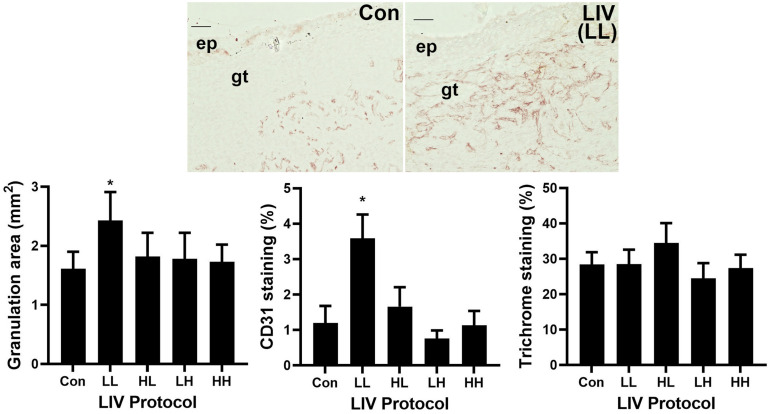
Parameter dependency of low-intensity vibration (LIV) for improving granulation and angiogenesis. Mice either received one of four protocols of whole-body LIV: 0.3 g at 45 Hz (LL), 0.3 g at 90 Hz (LH), 1.0 g at 45 Hz (HL), 1.0 g at 90 Hz (HH) or sham control treatment (Con) for 30 min per day, starting the day of wounding for 5 days per week. Left: Granulation tissue thickness was measured as the area of granulation tissue divided by the distance between wound edges in hematoxylin and eosin stained sections from center of day 10 wounds. Center: Angiogenesis was measured as percent area stained with antibody against CD31 in sections from center of day 10 wounds. Top: Representative images of CD31 stained sections; ep, epithelium; gt, granulation tissue; scale bar = 50 μm. Right: Collagen deposition assessed as percent area stained blue in Trichrome stained sections of center of day 10 wounds. Two wounds from each of four mice were analyzed for each assay (*N* = 8 total for each assay). *Mean value significantly different from that of Con, *P* ≤ 0.05.

Similar to the wound closure measurements, the LL protocol was the only LIV protocol to increase granulation tissue area on day 10 post-injury compared to sham controls (bar graph in Figure 2, example images in Figure 1). The other protocols produced no significant change in granulation tissue area. In addition, only the LL protocol induced a robust increase in angiogenesis on day 10 as assessed by CD31 staining compared to sham controls (Figure 2). The other protocols produced no significant change in angiogenesis. In short, the LL protocol improved angiogenesis and granulation tissue formation in diabetic mice, whereas the other protocols had no effect.

### LIV Enhances Wound IGF-1 Levels in a Parameter Dependent Fashion

**FIGURE 3:**
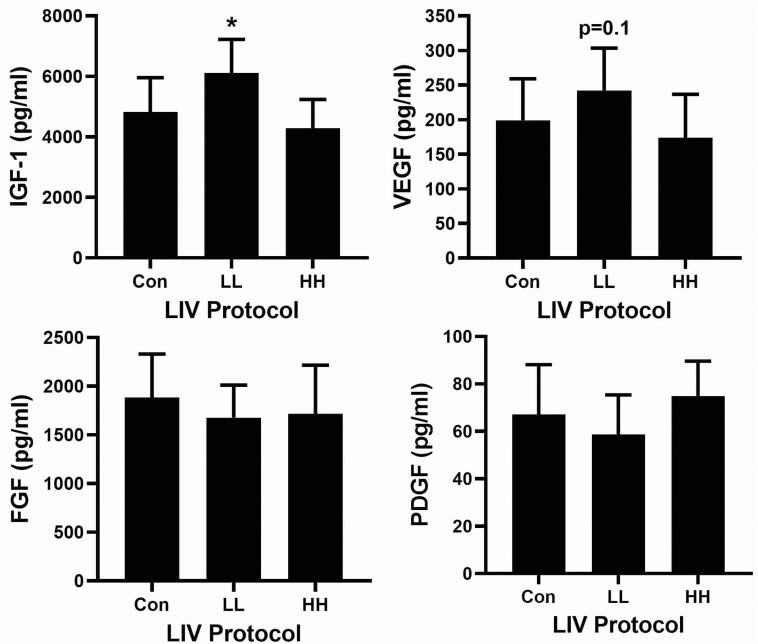
Parameter dependency of low-intensity vibration (LIV) for increasing IGF-1 in wounds. Mice either received one of two protocols of whole-body LIV: 0.3 *g* at 45 Hz (LL), 1.0 g at 90 Hz (HH) or sham control treatment (Con) for 30 min per day, starting the day of wounding for 5 days per week. Protein levels of IGF-1, VEGF, FGF-b, and PDGF-bb measured in homogenates of day 10 wounds using ELISA. Two wounds from each of four mice were analyzed for each assay (*N* = 8 total for each assay). *Mean value significantly different from that of Con, *P* ≤ 0.05.

Associated with the accelerated re-epithelialization and dermal healing, the LL protocol significantly increased levels of IGF-1 and tended to increase levels of VEGF in wounds on day 10 post-injury compared to sham controls (Figure 3). The other protocols did not alter wound levels of IGF-1 or VEGF. In addition, none of the LIV protocols altered wound levels of either bFGF or PDGF-bb. Thus, LIV induces specific growth factors during wound healing in a parameter dependent fashion.

## Discussion

Despite the escalating socioeconomic impact of diabetic wounds, effective treatments remain elusive. In this study, we sought to determine the parameter dependency of a novel therapeutic approach to improve diabetic wound healing using whole-body LIV. The major finding of this study is that only LIV with relatively low frequency (45 Hz) and low acceleration levels (0.3 g) improve wound healing in diabetic mice. Compared to non-vibrated control mice, such LIV treatment increased granulation tissue formation and angiogenesis, and accelerated closure and re-epithelialization. In contrast, LIV with higher frequency (90 Hz) and/or higher acceleration levels (0.6 g) tended to impair wound closure and had little to no effect on angiogenesis or granulation tissue formation. Thus, LIV does indeed exhibit parameter-dependent effects on wound healing.

The results of the current study are consistent with our previous study, in which we reported that LIV with 45 Hz frequency and 0.4 g acceleration increased angiogenesis, granulation tissue formation, and re-epithelialization (Weinheimer-Haus et al., 2014). This protocol was similar to our LL protocol (45 Hz frequency, 0.3 g acceleration). In addition, data in the present study demonstrates that protocols with higher frequency and/or acceleration do not improve skin healing. Related studies on the effect of LIV with similar frequency (47 Hz) and acceleration (0.2 g) on pressure ulcers (3.15 min treatments per day) demonstrated that LIV can improve healing of stage I pressure ulcers in elderly patients compared to standard care (Arashi et al., 2010). Similar LIV treatment for 15 min per day also reduced progression of pressure induced deep tissue injury associated with downregulation of matrix metalloproteinase-2 and -9 activity in rats (Sari et al., 2015).

A number of studies have focused on the effects of vibrations on skin blood flow in both rodents and humans, which could influence wound healing along with other physiological processes. LIV with frequency of 47 Hz and unknown acceleration increased skin blood flow measured by intravital microscopy of the mouse ear in what appears to be a nitric oxide dependent manner (Nakagami et al., 2007; Ichioka et al., 2011). A series of studies has also shown that vibrations can increase skin blood flow as measured by laser Doppler flowmetry when vibrations were applied to the forearm or lower leg in healthy human participants or diabetic patients (Lohman et al., 2007; Maloney-Hinds et al., 2009; Johnson et al., 2014). These latter studies used frequencies between 30 and 50 Hz and accelerations between 6 and 7 g, and found no difference in skin blood flow response between 30 and 50 Hz protocols (Maloney-Hinds et al., 2008). These protocols may be considered high-intensity vibration, since LIV is typically considered to utilize accelerations <1.0 g. We performed a similar experiment using LIV with frequency of 30 Hz and accelerations of 0.4 g and found that LIV signals also increase lower leg skin blood flow in healthy human participants but this effect only lasts while LIV is applied (Tzen et al., 2018). A recent study compared LIV protocols with frequencies of 35 and 100 Hz and an amplitude of 1 mm and found that the 100 Hz protocol produced larger increase in skin blood flow over the first metatarsal head (Zhu et al., 2020). Finally, recent systematic reviews have provided additional evidence that vibration can increase blood flow in the lower extremities in diabetic patients (Sa-Caputo et al., 2017; Gomes-Neto et al., 2019). These studies support the translation of LIV into a treatment that can improve wound healing in human patients.

Limitations of this study include the small number of parameters tested; two frequencies and two accelerations. However, our finding that only the 45 Hz, 0.3 g protocol improved wound healing narrows the solution space for pro-healing parameters. The parameters were measured at the surface of the vibrating plate; the actual vibrations experienced by the wound could be different and would be extremely difficult to measure. In addition, although we have identified growth factors that are increased by LIV and are associated with angiogenesis, granulation tissue formation and closure, the precise mechanisms involved remain to be elucidated. Along the same lines, although we assessed effects of LIV on re-epithelialization, granulation and angiogenesis, more detailed analysis of the effects of LIV on wound cells, including keratinocytes, fibroblasts, endothelial cells and inflammatory cells awaits further study.

In summary, our findings demonstrate parameter-dependent effects of LIV for improving wound healing and only LIV with 45 Hz frequency and 0.3 g acceleration levels increased angiogenesis, granulation tissue formation and re-epithelialization associated with increased wound levels of IGF-1. Importantly, the LIV protocol could easily be translated to clinical trials for diabetic patients with chronic wounds.

## Data Availability Statement

The raw data supporting the conclusions of this article will be made available by the authors, without undue reservation.

## Ethics Statement

All procedures involving animals were approved by the Animal Care Committee at the Jesse Brown Veterans’ Affairs Medical Center.

## Author Contributions

RR helped design the study, performed experiments, and wrote the manuscript. OB, RK, and TK helped design the study and write the manuscript. All authors contributed to the article and approved the submitted version.

## Conflict of Interest

TK has a patent pending on the application of vibration for therapeutic treatment for tissue repair “Method and system for physical stimulation of tissue” (#2013/0165,824). The remaining authors declare that the research was conducted in the absence of any commercial or financial relationships that could be construed as a potential conflict of interest.
